# CryoEM structures of evolved Family B DNA polymerase bound to template-primer substrates

**DOI:** 10.1063/4.0001170

**Published:** 2025-10-27

**Authors:** Millie M. Georgiadis, Nicole Leal, Steven A. Benner

**Affiliations:** 1 Indiana University School of Medicine, Indianapolis, IN, USA; 2 Foundation for Applied Molecular Evolution, Alachua, Florida, USA

## Abstract

The cryoEM revolution has made it possible to determine structures of many macromolecules of interest that have remained intractable to crystallographic analysis. But, despite the many advances in instrumentation and software, complexes with molecular weights that are less than 100 kDa remain on the small side for cryoEM. Particularly challenging are dynamic proteins that are undersized such as bacterial and archeal replicative DNA polymerases. A long-standing interest in my laboratory has been to provide a structural basis for understanding how laboratory evolved polymerases adapt to enable replication of unnatural nucleic acid. It has been necessary to create polymerase variants to replicate unnatural nucleic acid because the process of natural evolution has conferred very narrow selectivity of DNA polymerases for natural nucleic acid substrates. In our inaugural study, we found that a small change in a hydrophobic core of an evolved Klentaq polymerase capable of replicating Z:P pairs allowed the polymerase to attain a larger range of motion than possible in the parent polymerase. With the goal of structurally characterizing enzymes that are capable of replicating additional unnatural base pairs, we have pursued cryoEM analysis of a family B polymerase that replicates both S:B and Z:P pairs, which were created by Benner laboratory (FfAME). To date, we have established that cryoEM can be used successfully to obtain 2.8- 3 Å structures of nucleic acid complexes with a family B DNA polymerase (∼90 kDa) and that the conformations observed in our cryoEM structures differ from those observed in crystal structures of related enzymes. Future studies will include comparative structural and molecular dynamics analyses of both natural and unnatural DNA substrates bound to this enzyme.

